# A rare presentation of primary cardiac myxofibrosarcoma: Case report and literature review

**DOI:** 10.1002/cnr2.2033

**Published:** 2024-04-10

**Authors:** Sepideh Soltani, Maryam Garousi, Elahe Mirzaee, Sogol Koolaji, Hengameh Nazari, Sepideh Emami, Ali Zare Mehrjardi, Amir Mohammad Arefpour

**Affiliations:** ^1^ Department of Radiation Oncology, School of Medicine Iran University of Medical Sciences Tehran Iran; ^2^ Non‐communicable Diseases Research Center, Endocrinology and Metabolism Population Sciences Institute Tehran University of Medical Sciences Tehran Iran; ^3^ Department of Radiology Isfahan University of Medical Sciences Isfahan Iran; ^4^ Department of Cardiology, Firoozgar Hospital, School of Medicine Iran University of Medical Sciences Tehran Iran; ^5^ Department of Pathology, Firoozgar Hospital Iran University of Medical Sciences Tehran Iran

**Keywords:** brain metastasis, cardiac MRI, case report, hemoptysis, primary cardiac myxofibrosarcoma

## Abstract

**Background:**

Primary cardiac myxofibrosarcoma is a rare and aggressive malignancy, with the majority of approaching strategies relying on case reports. This article provides insights into its diagnosis and treatment.

**Case Presentation:**

This paper presents the case of a 40‐year‐old man with sudden onset hemoptysis, leading to the diagnosis of primary cardiac myxofibrosarcoma. Treatment involved open‐heart surgery to excise the left atrium tumor, followed by 6 cycles of adjuvant chemotherapy. Unfortunately, brain metastasis developed, leading to the patient's death 1 year after initial diagnosis.

**Conclusion:**

Primary cardiac myxofibrosarcoma remains a clinical challenge with an unfavorable prognosis. Early diagnosis through advanced imaging is crucial, and research is needed to explore innovative treatments. This case underscores the complexities of managing this rare cardiac malignancy and highlights the necessity for ongoing investigations to enhance patient outcomes.

## 1 INTRODUCTION

Primary cardiac tumors are exceptionally rare, with autopsy findings indicating an incidence of about 0.02%.^1^ Among these, about 75% are benign, primarily myxomas, whereas the remaining 25% consist of malignant entities, various types of sarcomas, and, less frequently, lymphomas.^2^

Myxofibrosarcoma predominantly occurs in the extremities of elderlies^3^ and is one of the rarest primary cardiac sarcoma subtypes^4^ with nonspecific primary symptoms.^5^

After reviewing multiple articles, it is clear that there is no widely agreed‐upon approach for primary cardiac myxofibrosarcoma, and our understanding largely relies on data from case reports.

In this article, we aim to present a case of a 40‐year‐old man with cardiac mass and the distinctive manifestation of sudden onset hemoptysis. This case contributes valuable insights into the diagnosis and treatment challenges of primary cardiac myxofibrosarcoma.

## 2 CASE PRESENTATION

**FIGURE 1:**
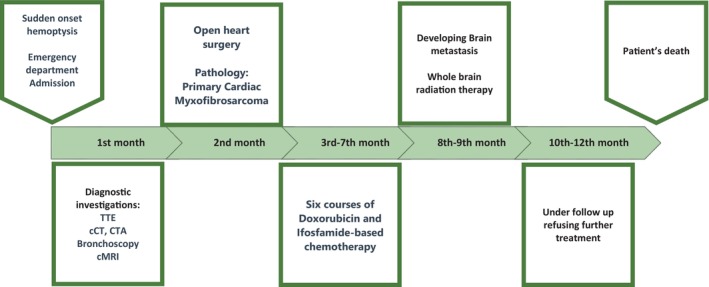
Case presentation timeline. cCT, cardiac CT; cMRI, cardiac magnetic resonance imaging; CTA, computed tomography angiography; TTE, transthoracic echocardiography.

A 40‐year‐old man was admitted to the emergency department of Firoozgar Hospital (Tehran, Iran) in July 2022 following sudden, profuse coughing of bright red blood while cycling, prompting him to seek medical attention immediately (see case presentation timeline, Figure 1).

At presentation, he was conscious and stable, with only mild palpitation but no other symptoms like chest pain, shortness of breath, cough, or wheezing.

He had a 10‐pack‐year smoking history and no significant medical or surgical history or medication use. He gave no history of dyspnea, chest discomfort, chronic cough, recent weight loss, fever, or fatigue.

On physical examination, vital signs were within normal range with a heart rate of 100 beats/min and oxygen saturation of 96% in room air; on cardiopulmonary assessment, decreased breath sounds on both lung bases, with cardiac loud S1 murmur raised concerns for potential underlying severe cardiac pathology; no lower extremity edema was evident.

His electrocardiogram (ECG) showed a sinus rhythm, with laboratory findings of normal D‐Dimer and NT‐proBNP levels and no evidence of inflammatory syndrome, anemia, renal, or hepatic abnormalities. Following stabilization, additional investigations began. His transthoracic echocardiography (TTE) showed an average left ventricular ejection fraction of 55% with normal left ventricular size, the presence of a large 75 × 28 mm mass in the left atrium (LA) extending into the left ventricle, mild mitral regurgitation, and moderate to severe functional mitral stenosis due to protruding LA mass.

His chest computed tomography (CT) scan had no parenchymal or mediastinal abnormalities, and CT angiography (CTA) showed no evidence of pulmonary thromboembolism. To rule out intrabronchial abnormalities, a bronchoscopy was performed that revealed no pathologic findings.

**FIGURE 2:**
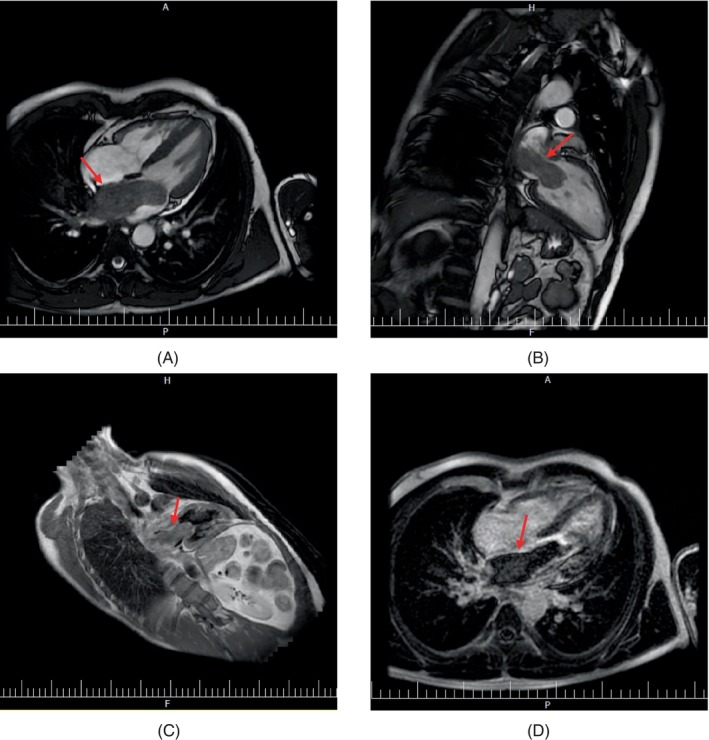
On SSFP cine images, the mass is isointense relative to normal myocardium, four‐chamber slice (A) long axis two chamber slice (B), on TI weighted double inversion recovery sequences, the mass is heterogeneous isointense hyperintense relative to normal myocardium (C) and on magnitude inversion recovery, late gadolinium enhancement images with myocardium nulling, the mass has heterogeneous enhancement (D), red arrow.

For further evaluation of the mass, cardiac magnetic resonance imaging (cMRI) was done, showing evidence of a large mobile LA mass (80 × 39 × 40 mm) extending into the left atrial appendage attached to the anterior mitral valve leaflet and protruding into the left ventricle during the diastolic phase, leading to induced mitral stenosis. On different sequences, the mass was heterogeneous relative to normal myocardium, which, due to MRI tissue characterization criteria, evidence was more suggestive of a malignant cardiac mass (Figure 2).

After normal diagnostic angiography of epicardial coronary arteries, open heart surgery was done, and the left atrium tumor measuring 80 × 60 × 20 mm was excised.

**FIGURE 3:**
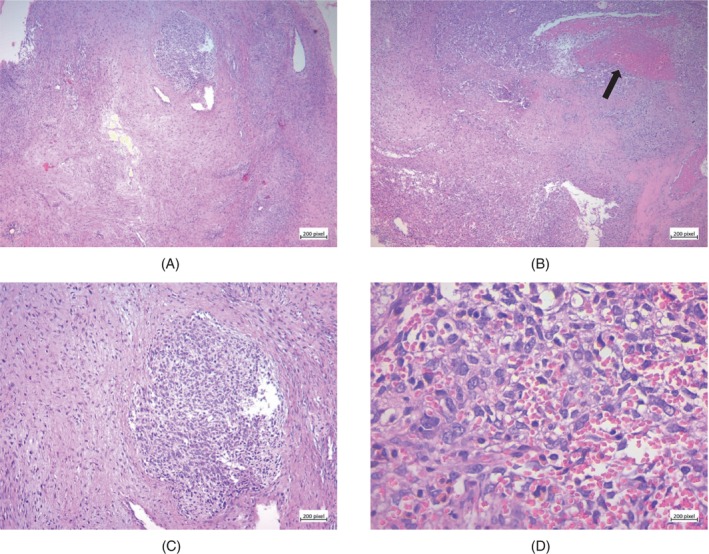
Spindle‐shaped tumor cells exhibit a complex, densely packed arrangement in a fibromyxoid background (Hematoxylin–Eosin, ×40 (A)). Cellular atypia with nuclear pleomorphism, extensive necrosis (black arrow), and hyalinization (Hematoxylin–Eosin, ×100 (B), ×100 (C), ×400 (D)).

**FIGURE 4:**
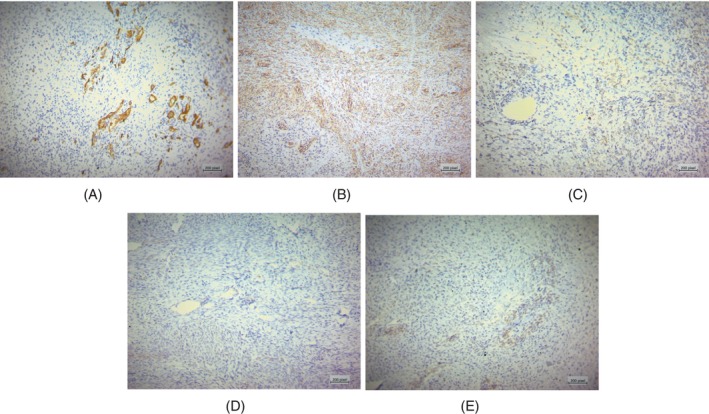
IHC results: CD34 × 100 (A), CD99 × 100 (B), Desmin × 100 (C), MyoD1 × 100 (D), TLE1 × 100 (E).

Pathologic examination showed neoplastic tissue composed of spindle cells and epithelioid cell proliferation in a fibromyxoid background. Cellular atypia with nuclear pleomorphism, extensive necrosis, hyalinization, and coarse curvilinear vessels were also seen (Figure 3). With these morphologic findings, the differential diagnosis included metastatic carcinoma, high‐grade leiomyosarcoma, pleomorphic rhabdomyosarcoma, and pleomorphic myxofibrosarcoma. IHC results ruled out metastatic carcinoma (negative CK), rhabdomyosarcoma (negative MyoD1), and leiomyosarcoma (negative desmin). CD34 highlighted a vascular pattern of the tumor. CD99 stain was cytoplasmic only and, therefore, nondiagnostic (Figure 4). Overall, these findings were consistent with pleomorphic myxofibrosarcoma.

As the surgical margins were not clear and the patient was not a candidate for a second surgery due to anatomic limitations, chemotherapy and radiotherapy were planned for him.

The patient underwent adjuvant chemotherapy, receiving a regimen that included intravenous administration of doxorubicin at a dose of 60 mg/m^2^ on days 1 through 3, intravenous administration of Ifosfamide at a dosage of 1500 mg/m^2^ on days 1 through 4, intravenous administration of Mesna at 900 mg/m^2^ per day on days 1 through 4, and daily subcutaneous doses of 5 mcg/kg of granulocyte colony‐stimulating factor for 10 days, initiated 24 h after the last dose of Mesna.^6^ This chemotherapy was administered every 21 days, with each course administered on an inpatient basis to facilitate close monitoring and provide supportive care throughout the treatment period.

**FIGURE 5:**
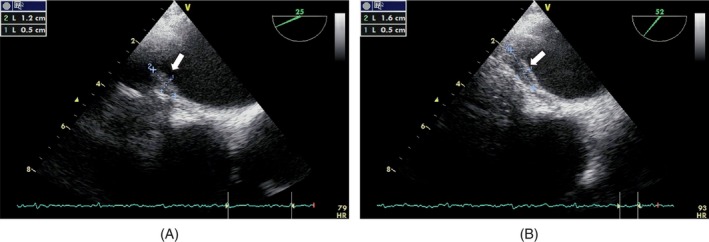
Linear mass density in the roof of LA in TEE (white arrow).

After six courses of chemotherapy, although TTE did not show any tumor, transesophageal echocardiography (TEE) indicated recurrence or remnant of LA neoplastic mass (Figure 5).

**FIGURE 6:**
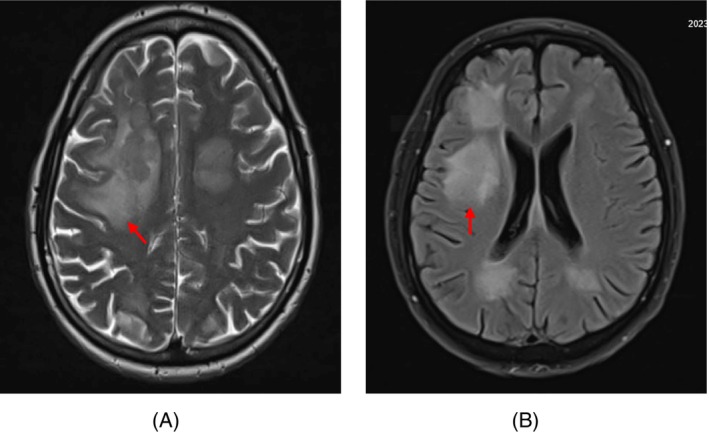
Multiple vasogenic regions of edema as high T2/FLAIR signal in both hemispheres of the brain. T2 sequence (A), T2/FLAIR sequence (B), red arrow.

The patient was scheduled for radiotherapy but had a sudden seizure. Brain MRI confirmed multiple intra‐axial metastatic lesions (Figure 6). Stereotactic biopsy was declined, and neurosurgery consultation deemed him unsuitable for brain surgery. Whole‐brain radiotherapy receiving a total dose of 30 Grays delivered in 10 fractions was done. There was no evidence of other sites of metastasis as seen in chest CT and abdominopelvic MRI. The patient remained stable and refused any further treatment. Five months later, he was admitted for loss of consciousness, and despite 10 days of ICU care, he died 12 months after the initial diagnosis.

## 3 DISCUSSION

The presented case involves a 40‐year‐old man diagnosed with primary cardiac myxofibrosarcoma, an exceedingly rare malignancy with limited literature that is summarized in Table 1. The unique aspect of sudden, massive hemoptysis as the primary symptom and the journey through diagnosis and treatment, along with the unexpected development of brain metastasis, adds to the significance of this case, making it a noteworthy clinical scenario.

**TABLE 1:** Presents compiled data from a thorough review of PubMed, Medline, and Web of Science databases, summarizing identified cases of primary cardiac myxofibrosarcoma in the English literature from 2018 to the present.

Study	Year	Patient	Clinical presentation	Diagnostic tools	Location	Size	Treatment and natural history (Respectively)
21	2018	18 M 13 F Mean = 41.87 ± 17.8	Most common: Dyspnea	Most common: TTE, TEE	Most common: Left atrium	Mean = 43.12 ± 16.18 mm	Most common: Sx → CTx → RTx → Local Recurrence
21	2018	F, 34 y	Headache, Dyspnea, Chest Pain	TTE	Left atrium	50 × 35 mm	Brain Met at presentation Sx (Primary Site) → NA
27	2019	F, 73 y	Dyspnea, Cough	CTA, TEE, TTE, cMRI, FDG‐PET CT, Gene Sequencing	Left atrium	46 × 32 mm	Sx → F/U × 8 m → Local Recurrence → CTx (Doxorubicin) × 3 → F/U × 24 m → Local Recurrence → CTx (Gemcitabine) × 3 → F/U
18	2020	F, 62 y	Dyspnea	TTE, TEE, cMRI, FDG‐PET CT	Left atrium	48 × 35 mm	Sx → CTx (Doxorubicin + Ifosfamide) → RTx → F/U
28	2020	F, 57 y	Dyspnea, Hemoptysis	TEE	Left atrium	45 × 35 mm	Sx → F/U
29	2022	F, NA	Dyspnea	TTE	Left atrium	NA	Sx (Myxoma Dx) → F/U × 6 m → Local Recurrence (3 masses) → Sx (MFS Dx) → F/U
30	2022	F, 72 y	Dyspnea, Chest Pain, Palpitation, Syncope	US	Left atrium	4 masses largest = 50 × 50 × 25 mm	Sx → F/U × 4 → Local Recurrence → F/U
11	2023	F, 61 y	Dyspnea, Palpitation	TTE, TEE, cCT, cMRI, FDG‐PET CT	Right ventricle	33 × 26 × 33 mm	Sx → CTx (Gemcitabine + Docetaxel)
31	2023	M, 65 y	Dyspnea, Cough	TTE, CTA, cCT, cMRI	Left atrium	50 × 28 × 28 mm	NA
32	2023	F, 75 y	Dyspnea, Edema	TTE, TEE, cCT, cMRI	Left atrium	2 masses largest = 68 × 43 mm	Sx → F/U × 3 m → Brain Metastasis and Death

Abbreviations: cCT, cardiac CT; cMRI, cardiac magnetic resonance imaging; CTA, computed tomography angiography; CTx, chemotherapy; Dx, diagnosis; F/U, follow‐up; F, female; FDG‐PET CT, fluorodeoxyglucose‐positron emission tomography; M, male; m, months; NA, not available; RTx, radiotherapy; Sx, surgery; TEE, transesophageal echocardiography; TTE, transthoracic echocardiography; US, ultrasonography; Y, years old.

The distinctive feature of this case lies in the atypical presentation of massive hemoptysis emerging as the primary symptom.

As mentioned by Tyeballi et al., cardiac tumors can manifest either as symptomatic entities or as incidental findings in the course of unrelated clinical presentations^7^ Also Dyspnea on exertion was the most common, whereas hemoptysis was among the less frequent presentations of primary malignant cardiac tumors.^8^

The identification of a heart murmur in the emergency room triggered further cardiac diagnostic investigations, and this highlights the role of history‐taking and physical examination in narrowing down the broad‐spectrum differential diagnosis of hemoptysis as a rare presentation of cardiac mass.^9^

Although the majority of primary cardiac tumors are benign,^10^ Early diagnosis and prompt treatment are crucial for enhancing patient survival in cases of malignancies, particularly in the context of MFS, because due to the absence of hallmark symptoms during the initial disease stages, these conditions are associated with a poor prognosis.^11^

Given the heterogeneous spectrum of cardiac masses, ranging from clots or vegetations to primary or metastatic malignancies, a consensus on the optimal diagnostic approach has yet to be established.^12^

According to an article by Lemasle et al., the current Cardiac imaging modalities are transthoracic echocardiography (TTE), transesophageal echocardiography (TEE), cardiac magnetic resonance (cMR), cardiac computed tomography (CT), and 18Ffluorodeoxyglucose positron emission tomography (18F FDG‐PET). TTE or TEE can make an initial assessment with other modalities used as a complementary tool.^13^

The diagnostic process started with a thorough evaluation using transthoracic echocardiography and cardiac magnetic resonance imaging (MRI). The transthoracic echocardiography revealed a large mass, prompting further investigation. Notably, cardiac MRI played a crucial role in confirming the malignant nature of the tumor, proving its high accuracy in distinguishing between benign and malignant cardiac lesions, aligning with findings from Pazos‐López et al.^14^

Recent research underscores the importance of 18F‐FDG PET/CT in distinguishing benign and malignant lesions and independently predicting survival through personalized decision‐making processes^15^ reviewing articles reveals a trend toward incorporating PET CT scans as part of the diagnosis, treatment planning, and follow‐up of primary cardiac tumors.

According to the latest(5th) WHO classification of thoracic tumors, primary cardiac sarcomas are categorized into Angiosarcoma, Leiomyosarcoma, undifferentiated pleomorphic sarcoma, and other sarcomas, including myxofibrosarcoma, synovial sarcoma, rhabdomyosarcoma, osteosarcoma, dedifferentiated liposarcoma, MPNST, and Ewing tumor.^16^

Myxofibrosarcoma is among the rarest subtypes of primary cardiac sarcomas that, according to reports, has predominantly been observed within the left atrium.^17^

Myxofibrosarcoma exhibits a broad range of cellularity and nuclear pleomorphism in the background of myxoid stroma; its characteristic pattern is curvilinear vascular arborization with necrosis indicating higher grades.^18^, ^19^ Although IHC markers do not help distinguish benign and malignant myxomas, they play a significant role in differentiating myxofibrosarcoma from other types of malignant tumors.^20^

In our case, a thorough review of surgical pathologic blocks, consistent with previous studies, confirmed the diagnosis. Negative IHC results for cytokeratin, desmin, and S100 ruled out sarcomatoid carcinoma, leiomyosarcoma, melanoma, and nerve sheath tumors.^21^

The subsequent phase of the case involved dealing with myxofibrosarcoma diagnosis, we followed the standard practice of surgical management and adjuvant chemotherapy, aligning with established methods supported by studies such as Chan et al. that emphasized the importance of surgical intervention, which resonated with our case, highlighting surgery's fundamental role in managing myxofibrosarcoma.^22^

Sun et al. analysis further supported the common use of surgery along with chemotherapy or radiotherapy in treating cardiac myxofibrosarcoma patients. The various systemic therapies used in their study, like anthracycline, imatinib, and ifosfamide, showcase the diverse approaches considered for these challenging cases.^21^

However, our case faced complications like anatomical limitations leading to incomplete surgery and the emergence of brain metastasis. In response, we shifted from systemic chemotherapy to brain radiotherapy, adapting our treatment plan to address these challenges.

Despite these adjustments, the overall prognosis for myxofibrosarcoma remains poor.^23^ given adverse features like incomplete surgery and the presence of an 80 mm mass with high‐grade features aligning with poor prognosis indicators.^21^, ^24^

Systemic treatments for primary cardiac sarcomas have seen limited progress due to the rarity of the condition and a lack of clinical trials.^24^ Metastatic MFS patients, reliant on standard chemotherapy, face limited options and poor outcomes.^19^ Özgü E et al. recent case report explores targeted therapies and the genomic landscape of myxofibrosarcoma (MFS). Studies revealed frequent alterations in TP53, CDKN2A/B, RB1, and ATRX genes. The integration of comprehensive genomic profiling in heavily treated, chemotherapy‐resistant MFS patients is proposed as a novel approach, providing potential alternative treatments. The report underscores the potential benefits of MEK inhibitors for individuals with activating RAF1 point mutations. This emphasizes the crucial role of personalized genomic insights in refining treatment strategies for the challenging realm of the MFS.^25^

The limited improvements in mortality over time highlight the systemic nature of the disease, emphasizing the need for a multidisciplinary approach and more robust therapies.^26^ While approaches, such as neoadjuvant chemotherapy and gene sequencing‐based targeted therapies, showed promising results, their broader applicability requires further investigation.^24^, ^27^

## 4 CONCLUSION

A substantially rare cardiac tumor case was presented in this paper, and the recently applied clinical approach in the literature was reviewed. This case report highlights the unique presentation of primary cardiac myxofibrosarcoma, emphasizing the rarity of hemoptysis as an indicator. The significance of early diagnosis through advanced imaging, particularly cardiac magnetic resonance imaging (cMRI), is underscored. The challenges faced, including incomplete surgical resection and brain metastasis, illuminate gaps in current therapeutic approaches where the prognosis for primary cardiac sarcomas remains poor. Our contribution emphasizes the need for ongoing research to explore innovative treatments beyond traditional interventions, such as neoadjuvant chemotherapy, gene sequencing‐based targeted therapies, and advanced radiation techniques, to improve outcomes for this rare cardiac malignancy.

## AUTHOR CONTRIBUTIONS

**Sepideh Soltani:** Writing – original draft (lead); writing – review and editing (lead). **Maryam Garousi:** Writing – review and editing (equal). **Elahe Mirzaee:** Data curation (equal); writing – review and editing (equal). **Sogol Koolaji:** Writing – review and editing (equal). **Hengameh Nazari:** Visualization (equal); writing – review and editing (equal). **Sepideh Emami:** Data curation (equal); visualization (equal). **Ali Zare Mehrjardi:** Data curation (equal); writing – review and editing (equal). **Amir Mohammad Arefpour:** Conceptualization (lead); supervision (lead); validation (lead).

## CONFLICT OF INTEREST STATEMENT

The authors have stated explicitly that there are no conflicts of interest in connection with this article.

## ETHICS STATEMENT

We obtained a written statement of informed consent from the patient for the publication of case details and the use of images. The case discussed in this manuscript does not include patient‐identifying information, nor does it report a new study that required IRB approval.

## Data Availability

The data supporting the findings of this study can be provided by the corresponding author upon a reasonable request.
